# Causal relationship between dietary habits and low hand grip strength: A Mendelian randomization study

**DOI:** 10.1097/MD.0000000000050178

**Published:** 2026-08-14

**Authors:** Enze Liu, Fengyu Sun, Dan Peng, Wantong Xu, Qinyu Zou, Xia Chen, Xiaoyong Wu

**Affiliations:** a Department of Radiology, The Affiliated Hospital of Jiangxi University of Chinese Medicine, Nanchang, Jiangxi, China; b Department of Radiology, The Second Xiangya Hospital, Central South University, Changsha, Hunan, China; c Department of Orthopedic Surgery, The Second Xiangya Hospital, Central South University, Changsha, Hunan, China; d School of Medical Imaging, Changsha Medical University, Changsha, Hunan, China.

**Keywords:** causal relationship, dietary habits, low hand grip strength, Mendelian randomization, reverse Mendelian randomization

## Abstract

A large number of studies have shown a close association between dietary habits and low hand grip strength. However, the presence of a causal connection between dietary habits and low hand grip strength remains unclear. All genome-wide association studies of dietary habits summary statistics were run in BOLT-LMM software (Broad Institute) using linear mixed models with 10 genetic principal components and genotyping array as covariates, and all phenotypes were averaged over multiple Food Frequency Questionnaires, adjusted for age and sex, and inverse normal transformed. The extracted single nucleotide polymorphisms explored the relationship between dietary habits and low hand grip strength by inverse variance weighted (IVW), Mendelian randomization-Egger (MR-Egger), and MR-pleiotropy residual sum and outlier. IVW estimates indicated that different dietary habits had a causal association with low hand grip strength, including drinking currently with various meals (beta: −0.432, 95% confidence interval [CI]: −0.669 to −0.194, *P*_IVW_ = 3.70E−04), semi-skimmed milk (beta: −0.825, 95% CI: −1.626 to −0.024, *P*_IVW_ = 4.30E−02), flora and benecol spread (beta: 0.210, 95% CI: 0.037–0.382, *P*_IVW_ = 1.80E−02), tablespoons of raw vegetables per day (beta: −0.361, 95% CI: −0.563 to −0.158, *P*_IVW_ = 4.80E−04), no dairy restrictions (beta: 1.223, 95% CI: 0.372–2.074, *P*_IVW_ = 4.90E−03) and no eggs, dairy, wheat, or sugar restrictions (beta: 1.027, 95% CI: 0.377–1.677, *P*_IVW_ = 2.00E−03). All significant MR results are false discovery rate-corrected. This MR investigation revealed that various dietary habits were causally linked to low hand grip strength. Further investigations into their relationships are necessary and will contribute to preventing low hand grip strength.

## 1. Introduction

Sarcopenia is a disease characterized by an age-related decrease in muscle mass, and an increasing number of studies suggest that muscle mass tends to decline by about 1% to 2% per year. Muscle strength decreases by 1.5% to 5.0% annually when individuals reach their mid-50s.^[1]^ Low hand grip strength is one of the core clinical manifestations of sarcopenia.

The bulk of the muscle is made up of protein and water, which may be thought to be significantly influenced by dietary habits. In recent years, research into the intricacies between dietary habits and sarcopenia has begun to be revealed. Numerous studies have shown that dietary habits can either have a positive or negative influence on sarcopenia. For example, Chun-De Liao discovered that protein-rich nutritional composition supplementation combined with resistance exercise training improves muscle gains and facilitates physical activity in older adults, which can slow down the process of sarcopenia.^[2]^ Meanwhile, Sakiko Abe found that medium-chain triglycerides could increase the strength and function of muscle in the elderly.^[3]^ Also, Yacong Bo suggests that the combined supplementation of whey protein, vitamin D, and vitamin E can significantly improve muscle mass and muscle strength in older adults with sarcopenia.^[4]^

Mendelian randomization (MR) makes full use of genetic information to test the causal relationship between phenotypes, allowing for existing unmeasured interferences, which has been widely used to address problems in epidemiology.^[5]^ There are 3 significant characteristics of MR: it uses publicly available genetic summary data to solve causal questions; it addresses multiple clinical inquiries across research areas; it provides prioritization of potential drug targets.^[6]^ MR establishes causal effects on outcomes by using genetic variants linked to the modifiable risk factor as proxy indicators, where genotype distribution from parent to child is random. Therefore, extraneous interference factors do not influence the relationship between genetic variations and outcomes.^[7]^ As a result, MR can offer an objective evaluation of the relationship between dietary habits and low hand grip strength.

Recently, a wide use of MR has been applied to investigate the potential link between dietary habits and diseases, such as diabetes,^[8]^ hypertension,^[9]^ and obesity.^[10]^ For the purpose of assessing the causal relationship between dietary habits and low hand grip strength in this study, we performed an MR analysis. In order to verify the earlier findings, we aimed to identify as many meaningful causal relationships between dietary habits and low hand grip strength as possible and to find new suggestions for future researchers.

## 2. Materials and methods

### 2.1. Data sources

**Figure 1.**
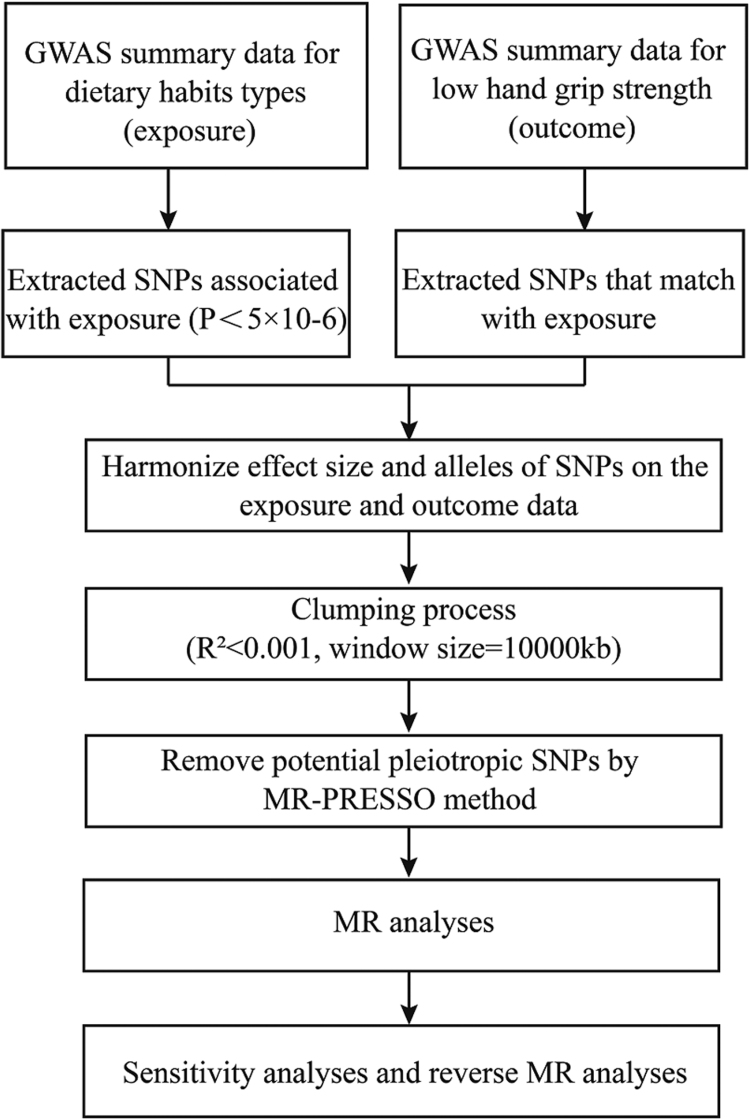
The working flow chart of this bidirectional Mendelian randomization study to explore the causal relationship between dietary habits and low hand grip strength. GWAS = genome-wide association studies, IV = instrumental variable, MR = Mendelian randomization, MR-PRESSO = MR-pleiotropic residual sum and outlier test, SNP = single nucleotide polymorphism.

The greatest genome-wide meta-analysis of dietary habits that has been published to date by JB Cole et al, “Comprehensive genomic analysis of dietary habits in UK Biobank identifies hundreds of genetic associations,” *Nature Communications* 2020, was used to compile genome-wide association studies (GWAS) summary statistics for dietary habits. Four assumptions should be met at the same time. First, single nucleotide polymorphisms (SNPs) should be strongly correlated with exposure. Second, SNPs should not correlate with the outcome directly. Third, are there any possible confounding factors related to SNPs? Last, there should be no genetic assortative mating in Figure 1. This is the design methodology for this study.^[11]^

### 2.2. Instrumental variable (IV)

Four selection criteria are used to choose suitable IVs in this study. First, the SNPs would be chosen as possible IVs that are linked with each dietary habit, with the locus-wide significance level (*P* < 5.0 × 10^−6^).^[12,13]^ Second, among the SNPs with *R*^2^ < 0.001 (clumping window size = 10,000 kb), the SNPs with the lowest *P* value were set aside. Third, horizontal pleiotropy is 1 important way that MR can deviate from the exclusion restriction assumption. In this work, to address the influence of horizontal pleiotropy, approaches like MR-Egger and MR-pleiotropy residual sum and outlier (MR-PRESSO) are used.^[14]^ We used the MR-PRESSO global test to detect horizontal pleiotropy. The pleiotropic and significant *P* value for each SNP was calculated using the MR-PRESSO outlier test to exclude horizontal pleiotropy. Last, it is appropriate to deduce the effect allele using the allele frequency data in order to harmonize palindromic SNPs.

### 2.3. Statistical analysis

Analyses were performed in R version 4.2.3 (The R Foundation for Statistical Computing, https://www.r-project.org) using 2-sample MR and Mendelian randomization packages. In this work, we used a variety of MR techniques, such as inverse variance weighting (IVW), MR-Egger regression, and MR-PRESSO, to determine whether there was a causal relationship between dietary habits and low hand grip strength. Potential pleiotropy bias was found using additional sensitivity studies.

The weight of each ratio in the IVW technique is equal to the inverse of the variance of the SNP-outcome correlation, which combines Wald ratios in fixed-effect meta-analysis. Sampling variation is the only reason why estimates from different studies differ from 1 another, which is an assumption made by the fixed effects meta-analysis. This corresponds to each SNP not showing any horizontal pleiotropy in the context of MR. The IVW technique would produce the most accurate results for fundamental causal estimates if all chosen SNPs were legitimate IVs. The IVW results would be impartial if horizontal pleiotropy did not exist.^[15]^ So, we can quantify the overall impact of diet on sarcopenia.

We also performed the MR-Egger regression to assess the potential for horizontal pleiotropy. Pleiotropy can make any SNP invalid, provided that it conforms to the instrument strength independent of direct effect assumption. Pleiotropy can be inferred if an MR-Egger regression’s intercept is not null.^[16]^ Otherwise, the MR-Egger regression’s outcome agrees with IVW.^[17]^

Furthermore, we used the MR-PRESSO test to examine pleiotropy.^[18]^ Tasks of MR-PRESSO are identifying horizontal pleiotropy, removing outliers to account for horizontal pleiotropy, and assessing.

We also used reverse MR analysis on the dietary habits, and it was discovered to be causally related to low hand grip strength in forward MR analysis. In the event that we can determine if dietary habits and low hand grip strength are causally related, we applied the *P* value threshold (*P* < 5.0 × 10^−6^) in this step, and the consistent methods with those of forward MR.

We have calculated the *F* statistic for each effective SNP using the algorithm to determine the IVs’ strength^[19]^:


$$F\;statistic={\left(\frac{{\hat{\beta }}_{X}}{se({\hat{\beta }}_{X})}\right)}^{2}$$

We assumed that there was no substantial weak instrumental bias if the corresponding *F* statistic was >10.

## 3. Results

### 3.1. Selection of instrumental variables

First, at the suggestive significance threshold *P* < 5.0 × 10^−6^, we discovered that SNPs are linked to dietary habits, including currently drinking with various meals, drinking semi-skimmed milk, no dairy restrictions, no eggs, dairy, wheat, or sugar restrictions, flora, and Benecol spreads, and tablespoons of raw vegetables per day. The *F* statistics for the SNPs included in this study all exceeded 10, indicating the absence of weak instrumental bias and thereby confirming the reliability of the research findings.^[11]^

We can then guarantee that the remaining IVs do not show any signs of horizontal pleiotropy by deleting pleiotropic SNPs that were detected by the MR-PRESSO outlier test and the MR-Egger regression (both MR-PRESSO global test *P* > .05 and MR-Egger regression *P* > .05).^[20]^

### 3.2. Associations of dietary habits and low hand grip strength

During the discovery stage, we used 5 MR approaches to research the causal relationship between each pair of dietary habits and low hand grip strength. It was found that 6 types of dietary habits were associated with low hand grip strength in at least 1 MR approach, including currently drinking with various meals, drinking semi-skimmed milk, no dairy restrictions, no eggs, dairy, wheat, or sugar restrictions, flora and Benecol spread, and tablespoons of raw vegetables per day. In Tables 1 and 2, certain statistics are displayed.

**Table 1:** Full positive results of MR estimates for the association between dietary habit types and sarcopenia.

Dietary habits (exposure)	Sarcopenia (outcome)	MR method	No. SNP	*F*	Beta (95% CI)	*P* value
Among_current_drinkers (drinks usually with meals yes + it varies vs no)	Low hand grip strength	IVW (MRE)	103	22.03	−0.432 (−0.669 to −0.194)	3.70E−04
		MR-Egger	103		−0.097 (−0.966 to 0.773)	.83
		Weighted median	103		−0.369 (−0.696 to −0.041)	2.70E−02
		Simple mode	103		−0.298 (−1.242 to 0.646)	.54
		Weighted mode	103		−0.298 (−1.196 to 0.599)	.52
Milk_type (semi-skimmed vs never)	Low hand grip strength	IVW (MRE)	20	26.31	−0.825 (−1.626 to −0.024)	4.30E−02
		MR-Egger	20		−0.433 (−2.891 to 2.026)	.73
		Weighted median	20		−1.512 (−2.666 to −0.358)	1.00E−02
		Simple mode	20		−1.756 (−3.807 to 0.294)	.11
		Weighted mode	20		−1.756 (−3.469 to −0.044)	.06
Never eat dairy vs no dairy restrictions	Low hand grip strength	IVW (MRE)	16	24.25	1.223 (0.372–2.074)	4.90E−03
		MR-Egger	16		3.790 (−0.065 to 7.644)	.07
		Weighted median	16		1.318 (−0.566 to 3.202)	.17
		Simple mode	16		1.321 (−1.917 to 4.559)	.44
		Weighted mode	16		1.255 (−2.071 to 4.581)	.47
Never eat dairy vs no eggs, dairy, wheat, or sugar restrictions	Low hand grip strength	IVW (MRE)	17	24.07	1.027 (0.377–1.677)	2.00E−03
		MR-Egger	17		3.026 (−0.080 to 6.132)	.08
		Weighted median	17		1.201 (−0.309 to 2.712)	.12
		Simple mode	17		1.187 (−1.450 to 3.824)	.39
		Weighted mode	17		1.187 (−1.422 to 3.796)	.39
Spread_type (flora + benecol vs never)	Low hand grip strength	IVW (MRE)	30	22.87	0.210 (0.037–0.382)	1.80E−02
		MR-Egger	30		0.366 (−0.065 to 0.796)	.11
		Weighted median	30		0.179 (−0.063 to 0.420)	.15
		Simple mode	30		0.272 (−0.235 to 0.779)	.3
		Weighted mode	30		0.116 (−0.382 to 0.615)	.65
Tablespoons of raw vegetables per day	Low hand grip strength	IVW (MRE)	126	21.57	−0.361 (−0.563 to −0.158)	4.80E−04
		MR-Egger	126		−0.307 (−1.052 to 0.437)	.42
		Weighted median	126		−0.309 (−0.608 to −0.009)	4.30E−02
		Simple mode	126		−0.309 (−1.072 to 0.453)	.43
		Weighted mode	126		−0.356 (−1.094 to 0.381)	.35

CI = confidence interval, IVW = inverse variance weighting, MR = Mendelian randomization, MRE = multiplicative random effects model, SNP = single nucleotide polymorphism.

**Table 2:** Full positive results of MR estimates for the association between sarcopenia and dietary habit types.

Sarcopenia (exposure)	Dietary habits (outcome)	MR method	No. SNP	*F*	Beta (95% CI)	*P* value
Low hand grip strength	Bread_type (white vs any other)	IVW (MRE)	31	22.3	0.021 (0.008–0.034)	1.30E−03
		MR-Egger	31		0.023 (−0.011 to 0.057)	.19
		Weighted median	31		0.018 (0.001–0.036)	4.30E−02
		Simple mode	31		0.038 (−0.005 to 0.081)	.09
		Weighted mode	31		0.035 (−0.008 to 0.078)	.12
Low hand grip strength	Bread_type (white vs whole meal whole grain + brown)	IVW (MRE)	31	22.3	0.020 (0.007–0.034)	2.50E−03
		MR-Egger	31		0.024 (−0.011 to 0.060)	.18
		Weighted median	31		0.018 (0.000–0.036)	.06
		Simple mode	31		0.029 (−0.015 to 0.073)	.21
		Weighted mode	31		0.027 (−0.015 to 0.070)	.22
Low hand grip strength	Bread_type (whole meal whole grain vs any other)	IVW (MRE)	31	22.3	−0.018 (−0.030 to −0.005)	6.30E−03
		MR-Egger	31		−0.032 (−0.068 to 0.004)	.09
		Weighted median	31		−0.012 (−0.031 to 0.006)	.2
		Simple mode	31		−0.010 (−0.054 to 0.034)	.65
		Weighted mode	31		−0.008 (−0.049 to 0.033)	.69
Low hand grip strength	Bread_type (whole meal whole grain vs white + brown)	IVW (MRE)	31	22.3	−0.023 (−0.035 to −0.011)	2.70E−04
		MR-Egger	31		−0.034 (−0.070 to 0.002)	.08
		Weighted median	31		−0.023 (−0.043 to −0.004)	1.70E−02
		Simple mode	31		−0.029 (−0.068 to 0.010)	.16
		Weighted mode	31		−0.029 (−0.069 to 0.012)	.18

CI = confidence interval, IVW = inverse variance weighted, MR = Mendelian randomization, MRE = multiplicative random effects model, SNP = single nucleotide polymorphism.

**Figure 2.**
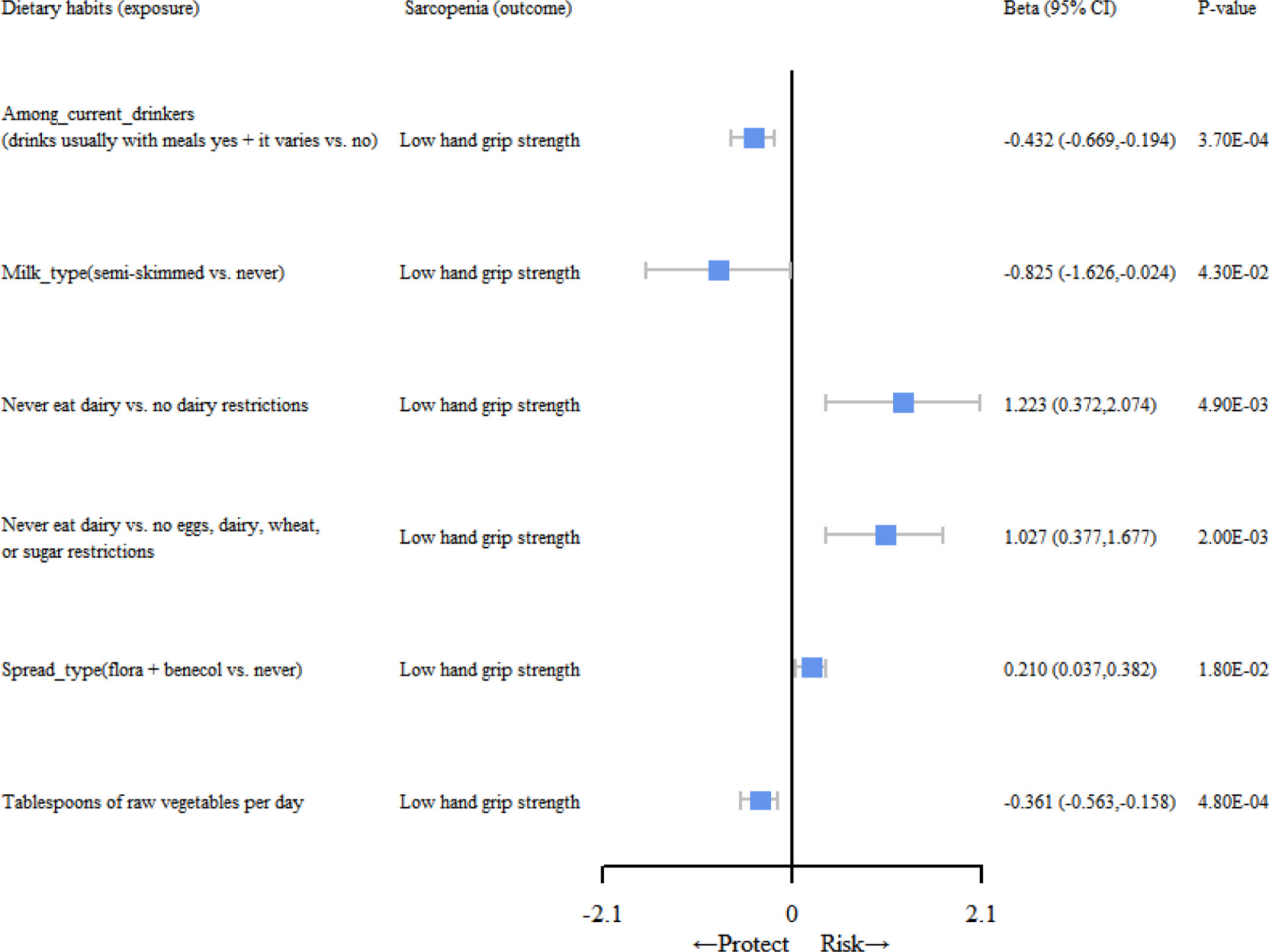
The forest plot showing MR results, which explores the causal effect of dietary habits and low hand grip strength. CI = confidence interval, MR = Mendelian randomization.

In Table S1, Supplemental Digital Content 1, Table S3, Supplemental Digital Content 2, and Table S5, Supplemental Digital Content 3, all the details of our research have been shown. As shown in Figure 2 and Table 1, after false discovery rate correction, the results of IVW analyses demonstrated genetically predicted causal effects that drinking currently with various meals has a significant protective impact against low hand grip strength, even after accounting for the related pleiotropy (beta: −0.432, 95% confidence interval [CI]: −0.669 to −0.194, *P*_IVW_ = 3.70E−04). Additionally, semi-skimmed milk (beta: −0.825, 95% CI: −1.626 to −0.024, *P*_IVW_ = 4.30E−02), flora and Benecol spread (beta: 0.210, 95% CI: 0.037–0.382, *P*_IVW_ = 1.80E−02), tablespoons of raw vegetables per day (beta: −0.361, 95% CI: −0.563 to −0.158, *P*_IVW_ = 4.80E−04), no dairy restrictions (beta: 1.223, 95% CI: 0.372–2.074, *P*_IVW_ = 4.90E−03), and no eggs, dairy, wheat, or sugar restrictions (beta: 1.027, 95% CI: 0.377–1.677, *P*_IVW_ = 2.00E−03) also had a protective effect on low hand grip strength, according to the IVW estimation.

### 3.3. Sensitivity analyses

All MR methods gave consistent estimates of effect for these causal relationships, and it boosts the certainty of a real association in Tables 3 and 4. We looked for any potential outlier variants that would have a significant influence on exposure by creating a scatter plot for the aforementioned statistics on the causal association, but no impact on the result.^[21]^ According to Table S6, Supplemental Digital Content 4, Table S7, Supplemental Digital Content 5, and Table S8, Supplemental Digital Content 6, the *F* statistics of genetic variants ranged from 21.57 to 26.51, which means that weak instrument bias was excluded.

**Table 3:** Sensitivity analyses for the association between dietary habit types and sarcopenia.

Dietary habits (exposure)	Sarcopenia (outcome)	No. SNP	Pleiotropy		Heterogenenity	
			MR-PRESSO global *P* value	MR-Egger *P* value	IVW test *P* value	MR-Egger *P* value
Among_current_drinkers (drinks usually with meals yes + it varies vs no)	Low hand grip strength	103	.1521	.434354972	.12964628	.12483552
Milk_type (semi-skimmed vs never)	Low hand grip strength	20	.4472	.743761388	.43062131	.37385789
Never eat dairy vs no dairy restrictions	Low hand grip strength	16	.993	.179687955	.99201407	.99904098
Never eat dairy vs no eggs, dairy, wheat, or sugar restrictions	Low hand grip strength	17	.9961	.193979185	.99555106	.99941174
Spread_type (flora + benecol vs never)	Low hand grip strength	30	.5326	.442273113	.51892653	.49798269
Tablespoons of raw vegetables per day	Low hand grip strength	126	.2400	.883983	.50076282	.47597401

IVW = inverse variance weighted, MR-Egger = Mendelian randomization-Egger, MR-PRESSO = Mendelian randomization pleiotropy residual sum and outlier, SNP = single nucleotide polymorphism.

**Table 4:** Sensitivity analyses for the association between sarcopenia and dietary habit types.

Sarcopenia (exposure)	Dietary habits (outcome)	No. SNP	Pleiotropy		Heterogenenity	
			MR-PRESSO global *P* value	MR-Egger *P* value	IVW test *P* value	MR-Egger *P* value
Low hand grip strength	Bread_type (white vs any other)	31	.2798	.896168278	.39058044	.34257247
Low hand grip strength	Bread_type (white vs wholemealwholegrain + brown)	31	.3114	.805276367	.38780218	.34212968
Low hand grip strength	Bread_type (whole meal whole grain vs any other)	31	.4558	.398003012	.70023642	.69027495
Low hand grip strength	Bread_type (whole meal whole grain vs white + brown)	31	.4152	.521542643	.79609421	.77497644

IVW = inverse variance weighted, MR-Egger = Mendelian randomization-Egger, MR-PRESSO = Mendelian randomization pleiotropy residual sum and outlier, SNP = single nucleotide polymorphism.

### 3.4. Reverse MR analysis

**Figure 3.**
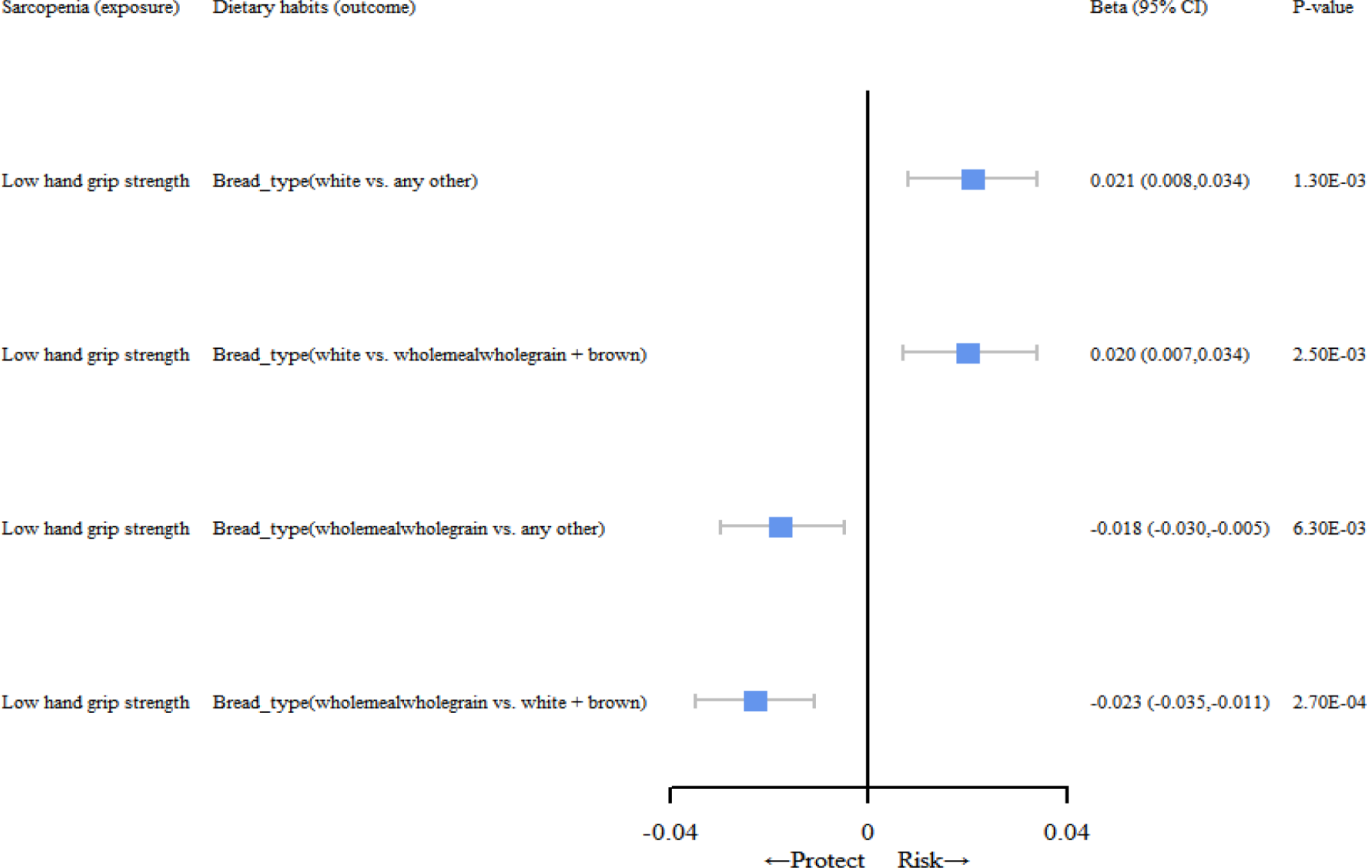
The forest plot showing reserve MR results, which explores the causal effect of low hand grip strength on dietary habits. CI = confidence interval, MR = Mendelian randomization.

All details of the reverse MR analysis were shown in Table S2, Supplemental Digital Content 7 and Table S4, Supplemental Digital Content 8. According to the results of the reverse MR analysis, as shown in Figure 3 and Table 2, after false discovery rate correction, we discovered evidence of a suggestive association between low hand grip strength and bread types, including white bread (beta: 0.021, 95% CI: 0.008–0.034, *P*_IVW_ = 1.30E−03), whole meal whole grain and brown bread (beta: 0.020, 95% CI: 0.007–0.034, *P*_IVW_ = 2.50E−03), whole meal whole grain bread (beta: −0.018, 95% CI: −0.030 to −0.005, *P*_IVW_ = 6.30E−03), and white and brown bread (beta: −0.023, 95% CI: −0.035 to −0.011, *P*_IVW_ = 2.70E−04).

### 3.5. Acknowledgments of MR study limitations

It is important to acknowledge the inherent limitations of the MR approach in interpreting these findings. While our analysis provides evidence supporting a potential causal relationship between the identified dietary habits and low hand grip strength, it represents an initial exploration rather than a definitive mechanistic explanation. The MR design infers causality based on genetic proxies and does not elucidate the specific biological pathways – such as those involving nutrient metabolism, inflammation, or the gut-muscle axis – through which these dietary factors may influence muscle health. Therefore, the mechanisms underlying the observed associations warrant further investigation through targeted experimental and longitudinal observational studies.

## 4. Discussion

We evaluated the possible causation between dietary habits and low hand grip strength in the current study by using MR analysis. Analyzing all the GWAS from the UK Biobank, we could identify 6 types of dietary habits that were causally associated with low hand grip strength.

In recent years, research into the nuanced relationship between dietary habits and sarcopenia has become more and more popular. According to Wen-Qing Xie research, caloric restriction can prevent the occurrence of sarcopenia in humans, especially in elderly adults. In this study, they describe the effects of caloric restriction on improving muscle protein synthesis, delaying muscle atrophy, regulating muscle mitochondrial function, maintaining muscle strength, promoting muscle stem cell regeneration and differentiation, and thus protecting against sarcopenia.^[22]^ Nevertheless, their method failed to reveal certain dietary habits related to calorie restriction. Currently, drinking with various meals, drinking semi-skimmed milk, having no dairy restrictions, no eggs, dairy, wheat, or sugar restrictions, using flora and Benecol spreads, and consuming tablespoons of raw vegetables per day – all of these dietary habits influence low hand grip strength. Furthermore, older adults experience eating and swallowing problems, dry mouth, taste loss, and anorexia, among other issues, causing “anorexia of aging” that affects their nutritional status, which can promote the occurrence of sarcopenia.^[23]^ Our findings might be a fresh starting point for research into certain dietary habits for preventing low hand grip strength.

Moreover, recent evidence underscores the importance of dietary patterns beyond single nutrients. A systematic review by Abiri et al emphasized that adherence to a Mediterranean diet – rich in fruits, vegetables, whole grains, and healthy fats – is associated with a lower risk of sarcopenic obesity, highlighting the synergistic effects of combined nutrients on muscle health.^[24]^ These findings suggest that holistic dietary approaches, rather than isolated supplements, may offer more sustainable strategies for low hand grip strength prevention.

Furthermore, protein supplementation has a significant effect on muscle mass in persons with sarcopenia, especially leucine supplementation, which can increase muscle mass and muscle strength, according to the study by Gielen et al.^[25]^ Additionally, the consumption of protein sources enhanced with this essential amino acid, or its metabolite beta-hydroxy-beta-methylbutyrate, is assumed to provide the greatest benefit in terms of the maintenance of muscle mass and function during aging.^[26]^

In light of our MR results, dietary interventions such as increasing raw vegetable intake and consuming semi-skimmed milk may exert protective effects against sarcopenia through multiple pathways, including anti-inflammatory actions, antioxidant supply, and gut microbiota modulation.^[27]^

Dietary habits are recognized to be a key determinant in shaping the composition and function of the gut microbiota.^[28]^ Overweight people may experience an altered microbial community structure and function, favoring propionic acid production during caloric restriction-induced weight loss.^[29]^ Toll-like receptor 9-deficiency in B cells has delineated cross-talk among toll-like receptor 9, adaptive immunity, and gut microbiota, leading to an enhanced inflammatory milieu and susceptibility to obesity and associated metabolic changes.^[30]^ Obesity stands as a public health challenge that poses a threat to the well-being of different people across the world. It may be a critical factor in many chronic diseases, especially sarcopenia.^[31]^

Furthermore, sarcopenia is closely related to obesity. When they occur concurrently, it is called sarcopenic obesity. Sarcopenic obesity is characterized by a concurrent decline in muscle mass and function, along with increased adipose tissue, which can lead to significant health consequences, including implications for mortality, comorbidities, and risk of developing geriatric syndromes.^[32]^ A growing body of evidence indicates that various dietary factors, such as appropriate calorie intake, macronutrient and micronutrient consumption, antioxidant nutrient intake, consumption of fruits and vegetables, and overall dietary quality, are linked to sarcopenic obesity.^[24]^

Therefore, from a practical perspective, dietary recommendations for low hand grip strength prevention should emphasize: adequate daily protein intake (≥1.2 g/kg/day) with an emphasis on leucine-rich sources; increased consumption of diverse vegetables and dairy products; incorporation of probiotic and prebiotic foods to support gut health; and maintenance of a balanced diet to avoid both undernutrition and obesity.^[33]^ Such strategies, supported by emerging evidence, can be integrated into public health guidelines to mitigate the burden of low hand grip strength in aging populations.

This study has several advantages. We used MR analysis to ascertain the relationship between dietary habits and low hand grip strength. Low hand grip strength is widely observed in elderly adults, and the process of its occurrence is controllable. We used a comprehensive GWAS meta-analysis to ensure the reliability of the tools used in the MR study. In this study, we used MR-Egger and MR-PRESSO to address the influence of horizontal pleiotropy. Furthermore, we evaluated the *F* statistic for each effective SNP to assess the strength of IVs.

There are several limitations in this study, though these limitations should be taken into consideration when interpreting the findings. It was not possible to conduct subgroup analyses for low hand grip strength because summary statistics rather than raw data were employed in the analysis. The SNP employed in the analysis did not meet the conventional GWAS significance level (*P* < 5.0 × 10^−6^), as more genetic variations need to be included as IVs in order to undertake sensitivity analysis and horizontal pleiotropy detection. Participants in this study were not restricted by their age and gender, and it was not possible to exclude the age and gender bias.

## 5. Conclusion

This MR study provides preliminary evidence of a causal relationship between specific dietary habits and low hand grip strength, highlighting the potential of dietary modifications as a strategy for prevention. The findings hold significance for guiding clinical nutritional interventions and underscore the value of genetic epidemiology in informing public health strategies for healthy aging. However, the exact mechanisms and clinical applicability of these associations require further validation through detailed mechanistic and longitudinal studies.

## Acknowledgments

The consortium’s release of dietary habits phenotypes of the T2DKP Summary Statistic was appreciated by the authors. The authors also wish to thank the authors of the GWAS summary statistics for low hand grip strength.

## Author contributions

**Conceptualization:** Dan Peng.

**Data curation:** Enze Liu.

**Formal analysis:** Enze Liu, Wantong Xu, Qinyu Zou.

**Funding acquisition:** Dan Peng, Xiaoyong Wu.

**Methodology:** Enze Liu, Fengyu Sun, Wantong Xu.

**Resources:** Dan Peng, Xia Chen, Xiaoyong Wu.

**Supervision:** Dan Peng, Xia Chen, Xiaoyong Wu.

**Validation:** Xiaoyong Wu.

**Visualization:** Enze Liu, Fengyu Sun, Dan Peng, Qinyu Zou, Xia Chen.

**Writing – original draft:** Enze Liu, Fengyu Sun.

**Writing – review & editing:** Fengyu Sun, Wantong Xu, Xiaoyong Wu.
















